# Short-Term Effects of Single-Session Split-Belt Treadmill Training on Dual-Task Performance in Parkinson's Disease and Healthy Elderly

**DOI:** 10.3389/fneur.2020.560084

**Published:** 2020-09-30

**Authors:** Nicholas D'Cruz, Jana Seuthe, Pieter Ginis, Femke Hulzinga, Christian Schlenstedt, Alice Nieuwboer

**Affiliations:** ^1^Neurorehabilitation Research Group, Department of Rehabilitation Sciences, KU Leuven, Leuven, Belgium; ^2^Department of Neurology, University Hospital Schleswig-Holstein, Christian-Albrechts-University (CAU) Kiel, Kiel, Germany

**Keywords:** dual-tasking, gait training, rehabilitation, freezing of gait, turning in place, constrained longitudinal data analysis

## Abstract

**Background:** Dual-tasking is challenging for people with Parkinson's disease and freezing of gait (PD+FOG) and can exacerbate freezing episodes and falls. Split-belt treadmill training (SBT) is a novel tool to train complex gait and may improve dual-task (DT) walking and turning.

**Objective:** To investigate the single-session effects of SBT on DT walking and DT turning performance in PD+FOG and older adults (OA), compared to regular treadmill training.

**Methods:** Forty-five PD+FOG and 36 OA participated in a single training session (30 min). They were randomized into one of four training groups: (A) SB75—steady belt speed ratio 0.75:1; (B) SB50—steady belt speed ratio 0.5:1; (C) SBCR—changing belt speed ratios between 0.75:1 and 0.5:1; and (D) Tied-Belt (TBT). Over-ground straight-line gait and an alternating turning in place task combined with a cognitive dual-task (DT) (auditory Stroop) were assessed pre- and post-training, and the following day (retention). Constrained longitudinal data analysis was used to investigate the training effects for all participants and for PD+FOG alone.

**Results:** DT gait speed improved at post-training for all groups (*p* < 0.001). However, SBT (SB50 and SBCR) led to larger post-training improvements compared to TBT, which were still visible at retention (SB50). For mean DT turning speed and Stroop response time while walking, only SBT groups showed significant improvements at post-training or retention. DT stride length, peak DT turning speed, and Stroop performance index while walking also showed larger gains in SBT compared to TBT. Results for PD+FOG alone showed similar effects although with smaller effect sizes.

**Conclusions:** A single session of SBT in PD+FOG and OA showed larger short-term effects on DT walking and turning compared to TBT. Cognitive DT performance was also improved in SBT, likely due to reduced cortical control of gait. These results illustrate the potential for SBT to improve DT during complex gait and possibly reduce fall risk in clinical and healthy populations.

## Introduction

Dual-tasking is defined as the concurrent performance of two or more (motor or cognitive) goal-directed tasks (1). Dual-tasking occurs frequently in daily life activities. However, performance of these tasks deteriorates with aging and cognitive decline (2). In Parkinson's disease (PD), due to motor automaticity deficits, increased cognitive control of gait is required (3), reducing available capacity to perform secondary tasks. Evidently, dual-tasking is more affected in PD in comparison to their healthy peers (4), and even more so in persons who show freezing of gait (FOG) (5) where cognitive impairment is greater (6). FOG is an episodic phenomenon that occurs in PD and can be characterized by the patients' “feeling of the feet being glued to the floor,” and are “unable to generate effective stepping despite the intention to progress forward and reach a destination” (7). Complex gait tasks—those that require locomotor adaptation—such as turning, are known to trigger FOG, and addition of a cognitive task worsens FOG (5, 8).

For both persons with PD and healthy older adults (OA), dual-tasking during walking impacts gait, as gait speed reduces (9, 10). Although the relationship between dual-task gait speed and fall risk is not yet established, reduced usual-gait speed has been associated with higher fall risk in OA (11) and together with FOG and previous fall status predicts fall risk in PD (12). Independent of gait speed, an experimental study using a reactive balance paradigm in PD showed that dual-tasking during protective stepping led to an increased incidence of falls, with little benefit of dopaminergic medication (13). Therefore, aiming to improve dual-task gait abilities is an important therapeutic goal in these populations, particularly during complex gait situations where fall risk is higher (14, 15). Although studies showing the effects of only motor training on dual-tasking in PD are scarce (16), improving motor and cognitive task performance separately or together through consecutive or integrated task training may be effective to improve dual-tasking (16, 17).

A recent systematic review (18) on the cognitive effects of acute aerobic exercise showed that short bouts of aerobic exercise improve interference control—a subcomponent of inhibition and closely linked to dual-task performance (19). Moderator analyses further revealed that effects were larger in age groups with poorer interference control such as preadolescent children and older adults. Based on the attentional capacity model, these effects of aerobic exercise modalities such as treadmill walking would boost attentional capacity and thereby dual-tasking performance. On the other hand, improving automaticity of a motor task may reduce its attentional demands, thereby freeing up resources for secondary task performance. Treadmill training may therefore improve dual-task gait through its cognitive as well as motor effects. However, given the task-specificity of motor learning, it is unclear whether these dual-tasking gains would transfer to complex gait situations and whether these gains would be similar in PD, thus necessitating this work.

A split-belt treadmill—where the belt of each foot can be driven independently of the other—offers the possibility to train motor adaptation during gait with varying amounts of complexity. In addition to the aerobic effects on cognition, the added variation and complexity of the motor task may lead to better gait automaticity and transfer to dual-tasking during complex gait situations. Although these effects have not yet been studied, the limited research in PD (20) has shown that patients are able to adapt to split-belt perturbations similarly to OA, albeit to a lesser extent (21, 22). Evidence of treadmill-driven gait adaptation effects on dual-tasking was shown in a study in OA and PD fallers which compared 6-weeks of regular treadmill walking to treadmill walking combined with a virtual reality (VR) based locomotor adaptation training (23). The VR offered some extra motor challenges and also increased cognitive task load. The authors found that while both groups similarly improved single task walking speed, walking under dual-task conditions improved more in the group that received the locomotor plus VR training. Although VR-based treadmill training was taken up well by people with FOG (24), it may be preferable to train tasks separately in this severely affected group, both in terms of motor and cognitive abilities, to avoid overloading available resources and optimize learning (25).

In this single-session proof-of-concept study, we used split-belt training (SBT) in comparison to tied-belt training (TBT) to investigate whether SBT had greater effects on dual-task performance in PD+FOG and OA. Our aim was to compare the effects of three levels of SBT complexity to tied-belt training (TBT) on dual-task performance during two over-ground conditions, namely straight-ahead gait and alternating turning in place. Based on the arguments presented above, we hypothesized that both TBT and SBT would improve dual-task gait speed, while only SBT would improve dual-task turning speed. Further, we expected similar effects on the cognitive task outcomes in both TBT and SBT during straight-line walking, while only SBT would improve cognitive performance while turning. As a secondary aim, we compared the various SBT groups based on retention of training effects, with a view toward making recommendations for longer-term repeated-training studies. We expected that the SBT condition with the largest difference in speeds would require greater adaptation and thus lead to the largest effect sizes. Overall, we anticipated that the results of this study would elucidate the effects of SBT on dual-tasking during complex gait as a stepping stone toward reducing fall risk in vulnerable populations.

## Methods

Recruitment and training of participants was conducted in two centers—namely the Department of Rehabilitation Sciences, KU Leuven, Belgium, and the Department of Neurology of the University Hospital Schleswig-Holstein, Christian-Albrechts-University of Kiel, Germany. The trial was pre-registered at ClinicalTrials.gov (NCT03725215), this study being a secondary analysis of the dual-task outcomes.

### Participants

**Figure 1:**
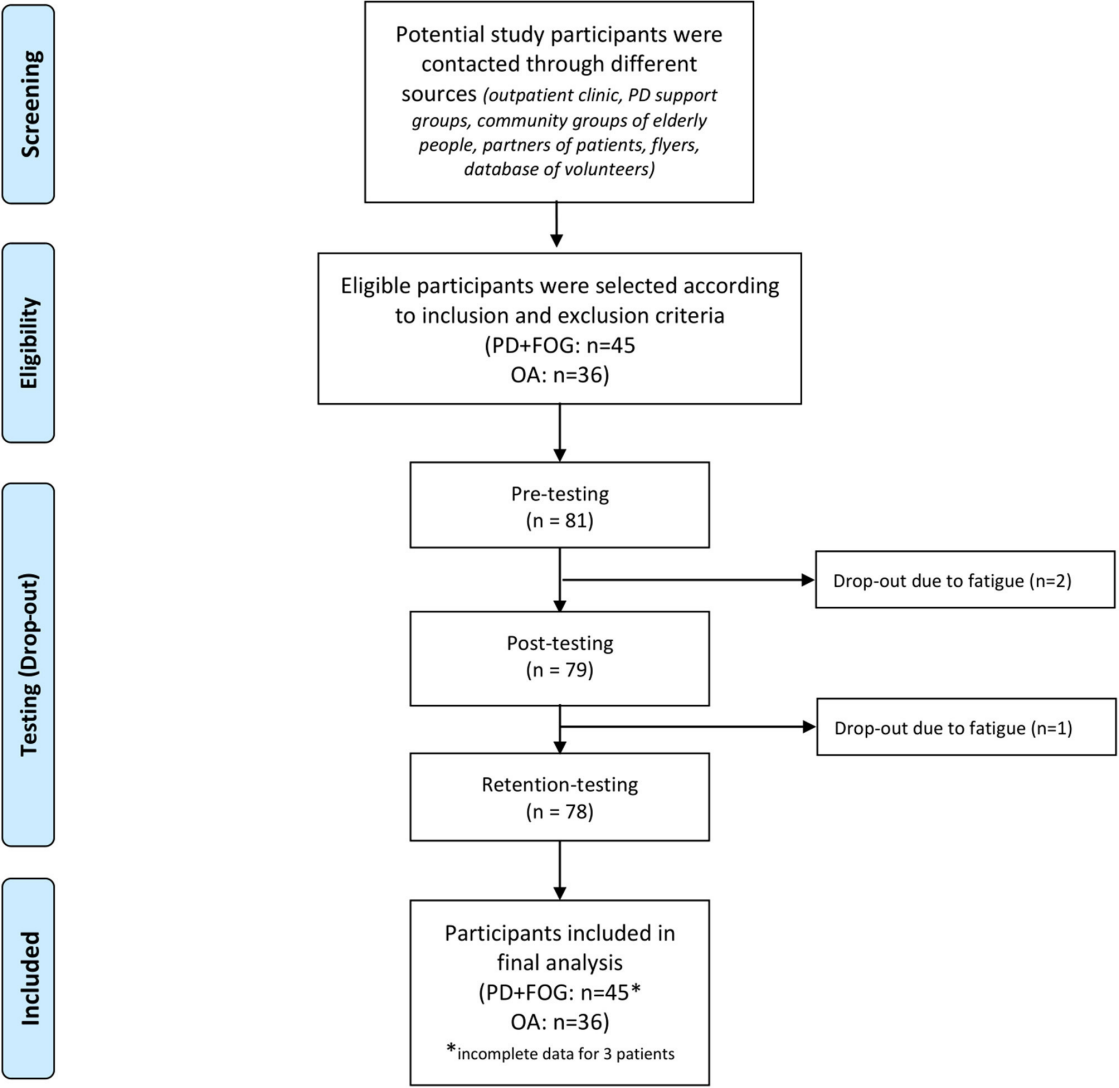
Flow diagram of recruitment process and study conduction; n, number of participants; PD+FOG, Parkinson's Disease with Freezing of Gait; OA, healthy older adults.

Forty-five persons with idiopathic PD as defined by the UK PD Brain Bank Criteria and FOG (as defined by the New Freezing of Gait Questionnaire—positive response to the question “did you experience Freezing episodes over the past month?”) (26) as well as 36 healthy OA were enrolled in the study between November 2017 and June 2019. Recruitment channels and numbers at each follow-up are given in Figure 1. Inclusion criteria were the ability to walk unassisted for at least 5 min (PD+FOG and OA) and to have stable antiparkinsonian medication and deep brain stimulator settings for at least 1 month to be included in the study (PD+FOG only). Exclusion criteria were neurological diseases apart from PD, treadmill exposure more than once a week, recent lower limb or back surgery (<6 months), cardiovascular risks for exercise assessed with the revised Physical Activity Readiness Questionnaire (27) (blood pressure regulator and/or antiplatelet usage permitted if no other risk), and cognitive impairment based on a Mini Mental State Examination (MMSE) score ≤ 24. The study protocol obtained ethical approval from the Ethics Committee for Research University Hospital/KU Leuven (Approval number: B322201734218) and the Ethics Committee of the University Hospital Schleswig-Holstein, Kiel (Approval number: D 454/13). Written informed consent was obtained prior to commencing screening in accordance with the declaration of Helsinki.

### Design and Intervention

This study utilized a non-blinded randomized parallel design. On inclusion, participants were block-randomized (block size: 4) within strata based on their Hoehn and Yahr disease stage (PD+FOG—four strata, stages I–IV) or their age group (OA—four strata from 45 to 85 years, each spanning 10 years) to receive one of four interventions. For the tied-belt training (TBT), both belts were set to the training speed. For the SBT conditions, one of the belts remained at the training speed while the other was slowed down. For one split-belt training paradigm, the slow belt was set to 75% of the training speed (SB75), based on previous work (28). Another split-belt training condition utilized a larger contrast, where the slow belt was set to half the training speed (SB50). The other condition was a switching-directed split-belt training where the speed of one belt alternated between 75 and 50% of the training speed (SBCR). The length of these switches was selected to have equal numbers of blocks at each speed with at least 20 strides before each switch (average of 37.5 s per switch). Blocked practice was used as it was previously shown to improve transfer to novel tasks compared with serial practice or random practice (29).

For all SBTs, the side to be reduced was the side with the longer step length during single-task over-ground walking, based on the best-side reduction principle that was previously shown to improve worse side step length in PD+FOG (28). Training speed was personalized to 85–100% self-paced single task over-ground walking speed, based on self-reported ability to train at that speed. This was assessed during a pre-training 2-min tied-belt treadmill familiarization trial. Participants were first exposed to the over-ground walking speed, and then speed was reduced in 5% steps, based on participant feedback. Over-ground walking speed was determined based on motion capture of at least five trials of straight line walking at a steady self-selected pace. The training was delivered in six blocks of 5 min, with a 1-min break in-between. For each training block in the split-belt condition, belts accelerated simultaneously (tied) until the target speed for the slower belt was achieved, while the faster belt continued accelerating until its target speed. Similarly, at the end of the block, the faster belt decreased speed until both belts were tied, after which both sides decelerated and came to a stop. Acceleration and deceleration was set to 1 m/s^2^ for participant safety.

If a participant indicated difficulty in completing the training, blocks were shortened in duration rather than in number and breaks were lengthened. Participants were not made aware of their particular training condition and were asked not to look down at the belts. Handrail use while training was discouraged to maximize motor learning (30), and participants wore a harness for safety without body weight support. Feedback was restricted to mentioning if foot placement was likely to cross the midline, and regarding the amount of time remaining in the training block.

### Assessments

Assessments were performed over two days at the same time of day and in the same order to standardize test timing in relationship to medication intake and circadian rhythm. Outcome measures were assessed thrice: pre-training (PRE) and post-training (POST) on day 1 and once on day 2 (retention—RET). To minimize the impact of split-belt after-effects on motor outcomes, post-training gait and turning assessments were separated from the training bout by at least 5 min of walking (~250 strides) on the treadmill as well as over-ground. PD+FOG were assessed and trained in the optimally medicated state. Demographics, cognitive, balance and clinical measures were assessed once as descriptors: Global cognition was measured with the Montreal Cognitive Assessment (MoCA) (31) and executive function with the Frontal Assessment Battery (32). Fall-related self-efficacy was assessed with the international version of the Falls Efficacy Scale (33) and balance performance with the Mini-Balance Evaluation Systems Test (Mini-BESTest) (34). Additionally, retrospective self-reported number of falls in the past 6 months was recorded. Clinical measures for PD+FOG included Hoehn and Yahr staging for randomization, the Movement Disorders Society sponsored revision of the Unified Parkinson's Disease Rating Scale motor examination part to assess disease motor burden (35), medication information from which the daily levodopa equivalent dose was calculated (36, 37) and the validated New Freezing of Gait Questionnaire (NFOGQ) for assessing FOG severity and impact (26).

### Outcome Measures

In this study, we only report on dual-task outcomes, which may more closely reflect daily-living gait (38). Based on previous work that investigated the reliability of dual-task outcomes in PD (39), primary outcome measures for the motor tasks were dual-task (DT) gait speed and mean DT turning speed and for the cognitive task was DT reaction time on the auditory Stroop test. Secondary outcomes for DT gait include outcomes previously shown to decrement on dual-tasking in PD (40) such as stride length, gait speed variability, stride length variability, step width and step width variability. Secondary outcomes for DT turning included mean speed variability, peak speed, peak speed variability and mediolateral jerkiness. Secondary outcomes for the cognitive dual task were response time variability, accuracy, and performance index (measure accounting for speed-accuracy tradeoff). For all variability measures, the coefficient of variation was used [CV = (SD/mean) ^*^ 100].

### Motor Tasks

#### Gait

Straight segments of over-ground self-paced gait over a 4-m walkway were captured using 3D motion analysis (Vicon Motion Systems, UK and Qualisys AB, Sweden), sampled at 100 Hz. Retroflective markers were placed on the heel and second toe and lateral malleolus bilaterally. Usual footwear was permitted for safety and comfort. No assistive devices were used. Ten walking trials were performed to capture at least 40 steps within the middle segment of the walkway at steady speed (41). Initial and terminal contact were determined based on the vertical velocity of the heel and toe markers (42). Spatiotemporal parameters of gait were calculated using custom scripts implemented in Matlab (version 2016b, The Mathworks, Natick, MA, USA).

#### Turning in Place

For the turning in place task, participants were asked to turn in place (360°) as quickly as possible for 1 min (43), alternating direction to avoid developing dizziness. Turning performance was captured with an inertial sensor placed on the lower back (OPAL™, APDM Inc., Portland, OR, USA). Angular velocity around the vertical axis was calculated from the gyroscope signal and changes in velocity direction were used to determine the start and end of each turn. Average and max angular velocity within each turn were calculated and averaged over turns to give the mean turning speed and peak turning speed. Integral of the squared time derivative of the accelerometer signal in the mediolateral direction was used to calculate turning jerkiness (44), a measure of turning fluidity. FOG episodes that occurred while turning were not excluded from the analyses, as this represents an ecological assessment of turning quality.

### Cognitive Task

The auditory Stroop (45) was used for the cognitive task as it is easy to deliver and record, with low possibility of learning effects over repeated testing. Further, quantification of response time as well as accuracy is possible, allowing more sensitive measures of task performance. For this task, the words “high” and “low” were presented in either a high pitch or a low pitch. The participant was asked to name the pitch that was heard, as quickly as possible. Random sequences of stimuli at varying intervals (spaced between 0.8 and 1.2 seconds apart to minimize cueing effects) were generated separately for the gait and turning task and for pre- and post-training. For the retention assessment, the pre-training sequences were re-used. Participant responses were recorded through a wireless headset and synced with the stimuli in an audio recording software (Audacity®, version 2.2.2, audacityteam.org). Response time and accuracy of responses were manually scored on replaying the recorded audio files. Performance index was calculated to take into account the speed-accuracy tradeoff as the percentage of correct responses per second. Instructions were neutral with regards to task prioritization (46)—participants were asked “to walk/turn and respond to the cognitive task at the same time.”

### Statistical Analysis

Demographic, cognitive, balance and clinical data were analyzed between training groups with one-way analysis of variance (Kruskal-Wallis for non-normal data) or chi-square likelihood tests for scalar or categorical variables, respectively. Constrained longitudinal data analysis (47) was used for the outcome analysis. This method is similar to an analysis of covariance, constraining groups to be similar at baseline as differences at baseline are due to chance and not of particular study interest. The Group^*^Time effect was dummy encoded to model changes from PRE compared to TBT (6 levels: 3 groups—SB75/SB50/SBCR ^*^ 2 time points—POST/RET). A linear mixed model was fit for each outcome measure as dependent variable and Age, Sex (2 levels—male and female), MoCA score, Center (2 levels—Leuven and Kiel), PD status (2 levels—PD and OA), Time (3 levels—PRE, POST, and RET) and the Group^*^Time effect (6 levels) as independent variables. An unstructured covariance matrix allowing for heterogeneity within each subgroup (PD/OA) of each training group (6 × 2 × 4 = 48 parameters) was used for the repeated effect of Time, after comparing the model fit of various covariance specifications (variance components, autoregressive, toeplitz) with the Akaike's Information Criteria. Satterthwaite approximation of the denominator degrees of freedom was used as the sample size was relatively small. Normality of residuals was visually assessed with histograms and Q-Q plots. Between and within-group effects over time were estimated from the model and effects sizes calculated using the t-statistic to d conversion.

$$\begin{array}{l} \text{Between}:\;d_{b}=\frac{2t_{b}}{\sqrt{df}}\text{ Within}:\;d_{w}=\frac{t_{w}}{\sqrt{df}} \end{array}$$

(48)

Effect sizes were interpreted as small (0.2–0.49), medium (0.5–0.79), or large (>0.8), in line with recommendations (49). A contrast statement with an F-test was used to test whether any group showed a change over time (within-group effect-−8 degrees of freedom) and whether there were differences between TBT and any SBT group (Group^*^Time effect - 6 degrees of freedom), before undertaking *post-hoc* testing to determine which group differed at which time point. Multiple comparisons were corrected with the false discovery rate (FDR) procedure (50). Sensitivity analyses were performed within the PD sub-group alone using the same methodology. Analyses were performed using SAS software, version 9.4 of the SAS System for Windows (SAS Institute Inc., Cary, NC, USA) and IBM SPSS Statistics for Windows, Version 26.0 (Armonk, NY: IBM Corp).

## Results

Forty-five PD+FOG (mean age: 68.5 years, SD: 10.2; 27% female) and 36 OA (mean age: 69.6 years, SD: 6.5; 44% female) were randomized into the four training groups. None of the participants had prior split-belt experience. Two PD+FOG randomized to SBT could not complete the training due to fatigue and dropped out after pre-training assessment while another PD+FOG dropped out after the post-assessment, also due to fatigue (Figure 1). The training groups were well-matched on demographic, cognitive, balance and clinical measures (*p* > 0.251) (Table 1). Further, training speed, training duration, handrail use and perceived intensity was also similar between-groups (*p* > 0.242) (Table 1). No adverse events related to the training were reported.

**Table 1:** Demographics, clinical measures (PD only) and training intensity for the four training groups.

	**TBT**	**SB75**	**SB50**	**SBCR**	***P***
**ALL (*****N*****)**	18	20	21	22	
Age (years)	67.38 (10.1)	68.1 (10.0)	69.33 (7.22)	71.09 (7.60)	0.556
Gender (% female)	16.7	35	38.1	45.5	0.251
MMSE (/30)	28.44 (1.88)	28.75 (1.11)	29.04 (1.20)	28.90 (1.37)	0.588
MOCA (/30)	25 (3.49)	26.26 (3.01)	25.66 (3.73)	25.77 (2.92)	0.713
FAB (/16)	16.05 (1.98)	16.26 (2.07)	16.57 (1.66)	16.72 (1.63)	0.659
6-M retrospective falls (*N*)	0 (1)	0 (2)	0 (2.5)	0 (0.3)	0.636
FES-I (/64)	22 (9)	18.5 (13.5)	20 (12.5)	22.5 (13.5)	0.601
Mini-BESTest (/28)	23.5 (7.5)	23.5 (8.3)	23 (8.5)	25 (6.5)	0.661
**PD (*****N*****)**	10	12	11	12	
Hoehn and Yahr (%-I/II/III/IV)	10/40/40/10	0/41.7/41.7/16.7	0/27.3/54.5/18.2	0/50/41.7/8.3	0.857
MDS-UPDRS part III motor (/132)	38.7 (21.6)	38.45 (16.1)	34.36 (11.0)	32.58 (12.1)	0.736
Disease duration (years)	11.65 (7.05)	13.2 (7.45)	14.36 (9.09)	12.33 (7.55)	0.87
LEDD	855.7 (353)	759.9 (294)	795 (323)	853 (417)	0.914
NFOG-Q (/28)	16.1 (6.53)	17.58 (6.11)	14.90 (6.10)	16.58 (5.07)	0.755
**Training intensity (ALL)**					
Training speed (m/s)	1.07 (0.20)	1.12 (0.21)	1.18 (0.30)	1.18 (0.26)	0.473
Training duration (min)	30 (0.87)	30 (0)	30 (0)	30 (0)	0.834
Training Shortened (Yes)	22.2%	10%	14.3%	18.2%	0.753
Handrail use (yes)	16.7%	20%	19%	9.1%	0.737
Borg Scale DURING (6–20)	12.05 (2.38)	11.45 (2.50)	11.05 (3.17)	11.6 (2.90)	0.743
Borg Scale POST (6–20)	13.17 (2.42)	12.5 (2.91)	11.95 (3.47)	12.59 (3.20)	0.685
VAS Mental Fatigue PRE (/10)	1.82 (1.36)	2.41 (2.33)	1.20 (1.40)	1.92 (1.90)	0.242
VAS Mental Fatigue POST (/10)	3.15 (2.10)	3.33 (2.83)	3.01 (2.62)	2.84 (2.19)	0.932
VAS Physical Fatigue PRE (/10)	2.64 (2.36)	2.65 (2.57)	2.08 (2.13)	2.54 (2.07)	0.854
VAS Physical Fatigue POST (/10)	3.95 (2.42)	3.71 (2.03)	3.78 (2.82)	3.42 (2.54)	0.928

*Means (SD) and one-way analysis of variance or chi-square likelihood test p-values are presented, except for FES-I, Mini-BEST and retrospective falls for which median (interquartile range) is reported along with a Kruskal-Wallis test p-value owing to non-normal distributions. PRE, Pre-training; DURING, halfway through training; POST, Post-training; MMSE, Mini Mental Status Examination; MOCA, Montreal Cognitive Assessment; FAB, Frontal Assessment Battery; 6-M Retrospective Falls, subjective recall of number of falls in last 6 months; FES-I, International version of Falls Efficacy Scale; Mini-BEST, Mini Balance Evaluation Systems Test; LEDD, daily Levodopa Equivalent Dose; NFOG-Q, new Freezing of Gait Questionnaire; VAS, Visual Analog Scale*.

### Combined Analysis Including All Participants

#### Motor Tasks

Significant Group^*^Time effects between TBT and SBT were seen on multiple aspects of DT gait including gait speed (*p* = 0.002), stride length (*p* < 0.001), step width (*p* = 0.025), gait speed variability (*p* = 0.017), and step width variability (*p* < 0.001) (Table 2). Although all groups showed within-group increases in gait speed and stride length, *post-hoc* tests revealed that increments were larger for SB50 (gait speed: p_−FDR_ = 0.001, stride length: p_−FDR_ = 0.001) and SBCR (gait speed: p_−FDR_ = 0.001, stride length: p_−FDR_ < 0.001) at POST; and SB50 (gait speed: p_−FDR_ = 0.02, stride length: p_−FDR_ = 0.014) and SB75 at RET (stride length only: p_−FDR_ = 0.05). Step width variability was significantly lower in SB50 at POST (p_−FDR_ = 0.001), but differences were lost at RET. *Post-hoc* tests were not significant for step width and gait speed variability.

**Table 2:** Within-group effects for each training group (PD+FOG and OA) from PRE to POST and PRE to RET, along with 95% Confidence Intervals (CI) and effect sizes (d_w_ from t statistic-paired).

	**Outcome**	**Time**	**TBT**		**SB75**		**SB50**		**SBCR**		Within/group*time	Group*time *post-hoc*
			**Estimate (95% CI)**	**d_**w**_**	**Estimate (95% CI)**	**d_**w**_**	**Estimate (95% CI)**	**d_**w**_**	**Estimate (95% CI)**	**d_**w**_**	***P***	
**GAIT**	DT gait speed (m/s)	PRE–POST	0.04 (0.00–0.08)	0.45^†^	0.07 (0.03–0.12)	0.82^†^	0.14 (0.11–0.18)	2.67^‡^	0.14 (0.09–0.18)	1.49^‡^	** <0.001**/ **0.002**	SB50 > TBT SBCR > TBT
		PRE–RET	0.09 (0.06–0.12)	1.48^‡^	0.13 (0.09–0.17)	1.56^‡^	0.16 (0.12–0.19)	2.84^‡^	0.15 (0.10–0.19)	1.75^‡^		SB50 > TBT
	DT gait speed CV (%)	PRE–POST	−0.6 (−2.8–1.61)	−0.14	0.94 (−0.9–2.80)	0.21	−2.5 (−5.1 to −0.0)	−0.52	−1.5 (−4.5–1.42)	−0.23	** <0.001/0.017**	NS
		PRE–RET	−3.7 (−5.5 to −1.8)	−1.00^†^	−4.2 (−6.2 to −2.3)	−1.07^†^	−5.5 (−7.3 to −3.7)	−1.12^‡^	−4.9 (−7.6 to −2.3)	−0.82^†^		NS
	DT gait stride length (mm)	PRE–POST	5.64 (−22–34.0)	0.08	35.2 (−3.6–74.1)	0.44^†^	96.4 (53.2–139)	1.20^‡^	103 (68.4–139)	1.20^‡^	** <0.001**/ ** <0.001**	SB50 > TBT SBCR > TBT
		PRE–RET	57.6 (31.3–83.9)	1.20^‡^	124 (88.5–160)	1.74^‡^	121 (85.6–156)	1.85^‡^	110 (63.5–157)	1.16^‡^		SB75 > TBT SB50 > TBT
	DT gait stride length CV (%)	PRE–POST	−1.1 (−3.0–0.81)	−0.43^†^	1.33 (−0.4–3.12)	0.35	−3.0 (−5.9 to −0.2)	−0.50	−2.6 (−5.7–0.43)	−0.38	** <0.001**/ 0.215	NS
		PRE–RET	−3.6 (−4.4 to −2.8)	−3.9^‡^	−3.9 (−6.0 to −1.7)	−0.91^†^	−4.5 (−7.0 to −1.9)	−0.85^†^	−3.9 (−7.0 to −0.7)	−0.60^†^		NS
	DT gait step width (mm)	PRE–POST	−5.2 (−8.1 to −2.4)	−1.2^†^	−2.1 (−4.0 to −0.2)	−0.53^†^	−1.2 (−4.2–1.67)	−0.24	−2.5 (−5.6–0.47)	−0.38	**0.001**/ **0.025**	NS
		PRE–RET	−3.1 (−9.7–3.43)	−0.26	−3.2 (−8.6–2.17)	−0.29	−6.1 (−12–0.35)	−0.48^†^	−0.7 (−8.2–6.65)	−0.04		NS
	DT gait step width CV (%)	PRE–POST	4.87 (1.20–8.53)	0.71^†^	3.67 (−1.2–8.55)	0.38	−8.8 (−13 to −4.5)	−1.07^†^	^†^(−6.3–1.91)	−0.23	** <0.001**/ ** <0.001**	SB50 < TBT
		PRE–RET	0.40 (−3.1–3.98)	0.06	−8.6 (−11 to −5.7)	−1.29^†^	0.13 (−2.6–2.93)	0.02	−3.2 (−6.7–0.21)	−0.41^†^		
**TURNING**	DT turning speed (°/s)	PRE–POST	3.59 (−2.6–9.82)	0.47	3.86 (−3.9–11.6)	0.27	6.01 (0.09–11.9)	0.50	9.87 (5.27–14.4)	0.97^‡^	** <0.001**/ **0.025**	NS
		PRE–RET	2.51 (−2.0–7.06)	0.36	7.83 (2.14–13.5)	0.77^†^	7.38 (0.12–14.6)	0.47	11.7 (5.35–18.0)	0.90^†^		NS
	DT turning speed CV (%)	PRE–POST	−0.9 (−1.9 to −0.0)	−0.49	−0.1 (−1.1–0.84)	−0.07	0.35 (−0.6–1.32)	0.22	−0.8 (−2.0–0.26)	−0.41	**0.052**/ 0.129	
		PRE–RET	−1.0 (−2.1 to −0.0)	−0.56	−0.4 (−2.1–1.22)	−0.15	−0.7 (−1.5–0.03)	−0.49	−0.6 (−2.9–1.68)	−0.11		
	DT peak turning speed (°/s)	PRE–POST	2.93 (−6.6–12.4)	0.20	12.1 (1.73–22.4)	0.67^†^	5.85 (−2.3–14.0)	0.34	8.27 (0.54–16.0)	0.44^†^	**0.002**/ **0.007**	NS
		PRE–RET	3.68 (−1.0–8.39)	0.47	8.80 (−0.4–18.0)	0.47^†^	12.6 (3.31–22.0)	0.63^†^	18.4 (9.50–27.4)	1.05^†^		SBCR > TBT
	DT peak turning speed CV (%)	PRE–POST	−1.2 (−2.8–0.28)	−0.47	0.92 (−0.4–2.28)	0.29	−0.1 (−1.1–0.86)	−0.06	0.08 (−0.9–1.16)	0.03	0.306 0.416	
		PRE–RET	−1.0 (−2.5–0.53)	−0.44	−0.0 (−1.4–1.34)	−0.02	−0.7 (−1.7–0.30)	−0.26	−0.1 (−2.2–1.99)	−0.02		
	DT turning jerkiness (m2/s5 * 100)	PRE–POST	2.37 (−0.5–5.25)	0.43	−0.1 (−1.7–1.48)	−0.03	2.15 (−0.2–4.58)	0.57	1.89 (−0.2–4.06)	0.40		
		PRE–RET	1.07 (−2.6–4.83)	0.15	−0.6 (−2.8–1.61)	−0.13	1.10 (−1.1–3.39)	0.26	2.32 (0.21–4.42)	0.44		
**GAIT STROOP**	Gait stroop response time (ms)	PRE–POST	−2.1 (−50–7.41)	−0.50	−1.2 (−48–24.3)	−0.17	0.26 (−31–36.9)	0.03	−3.2 (−62 to −2.1)	−0.50	**0.005** 0.287	
		PRE–RET	−1.0 (−41–21.4)	−0.16	−3.9 (−60 to −18)	−1.00^†^	−2.5 (−75–24.5)	−0.25	−3.6 (−70 to −2.1)	−0.48		
	Gait stroop response time CV (%)	PRE–POST	−1.3 (−2.6 to −0.1)	−0.53	−1.7 (−3.3 to −0.2)	−0.60	−0.8 (−3.0–1.30)	−0.19	−0.5 (−2.2–1.04)	−0.14	** <0.001** ** <0.001**	NS
		PRE–RET	1.69 (−0.3–3.74)	0.41	−2.0 (−3.6 to −0.4)	−0.60	−4.2 (−5.4 to −3.0)	−2.73^‡^	−0.1 (−1.6–1.34)	−0.04		SB75 < TBT SB50 < TBT
	Gait stroop accuracy (%correct)	PRE–POST	6.78 (3.38–10.1)	0.92^†^	0.84 (−0.4–2.10)	0.50	−6.0 (−10 to −1.7)	−0.73^†^	1.23 (−1.1–3.62)	0.33	** <0.001** ** <0.001**	TBT > SB75 TBT > SB50 TBT > SBCR
		PRE–RET	2.20 (0.25–4.15)	0.82^†^	3.63 (0.94–6.32)	0.62^†^	−1.8 (−9.5–5.77)	−0.13	4.25 (0.96–7.54)	0.69^†^		NS
	Gait stroop performance index (%/s)	PRE–POST	4.24 (0.36–8.12)	0.64^†^	5.61 (1.98–9.23)	0.70^†^	−6.1 (−11 to −1.0)	−0.60^†^	3.92 (−1.3–9.16)	0.39	** <0.001** ** <0.001**	TBT > SB50
		PRE–RET	−0.1 (−3.0–2.86)	−0.01	6.20 (2.97–9.43)	0.99^†^	0.10 (−9.8–10.0)	0.00	7.85 (1.59–14.1)	0.56^†^		SB75 > TBT
**TURNING STROOP**	Turning stroop response time (ms)	PRE–POST	−16 (−41–7.94)	−0.44	−36 (−78–5.99)	−0.41	−33 (−80–14.3)	−0.37	−65 (−107 to −23)	−0.62^†^	** <0.001** 0.616	
		PRE–RET	−3.6 (−77–3.76)	−0.45^†^	−72 (−101 to −43)	−1.19^‡^	−47 (−85 to −9.1)	−0.79^†^	−66 (−96 to −36)	−0.98^†^		
	Turning stroop response time CV (%)	PRE–POST	−1.4 (−3.5–0.60)	−0.40	−0.1 (−3.0–2.65)	−0.02	−1.7 (−3.6–0.06)	−0.47	−2.1 (−4.6–0.45)	−0.33	0.308 0.836	
		PRE–RET	0.63 (−2.2–3.49)	0.11	−1.0 (−3.1–1.07)	−0.23	−1.4 (−4.7–1.75)	−0.23	−1.2 (−3.7–1.32)	−0.22		
	Turning stroop accuracy (%correct)	PRE–POST	0.50 (−2.7–3.75)	0.07	1.94 (−0.8–4.77)	0.25	3.36 (0.30–6.42)	0.59	0.23 (−4.0–4.49)	0.03	0.310 0.727	
		PRE–RET	2.06 (−0.6–4.78)	0.43	0.91 (−1.8–3.62)	0.26	0.43 (−3.8–4.71)	0.05	1.36 (−2.3–5.05)	0.19		
	Turning stroop performance index (%correct/s)	PRE–POST	3.62 (−2.0–9.25)	0.33	5.44 (1.79–9.08)	0.63^†^	5.83 (1.23–10.4)	0.75^†^	4.51 (−2.0–11.0)	0.31	**0.002** 0.919	
		PRE–RET	4.74 (−0.0–9.55)	0.49	7.73 (2.47–13.0)	0.77^†^	5.55 (−0.1–11.2)	0.55	7.60 (2.58–12.6)	0.66^†^		

Effect size values are reported in the direction of the raw data, not in the direction of expected improvement. F-test probability values testing any within-group change from PRE as well as Group*Time interactions are also reported, along with post-hoc tests for which multiple comparison correction was performed with the False Discovery Rate method (FDR). Statistical significance indicated by^†^p_FDR_ < 0.05, p_FDR_ < 0.001 or **Bold** probability values.

For DT turning outcomes, significant interaction effects between TBT and SBT were found for mean (*p* = 0.025) and peak (*p* = 0.007) turning speed. Although *post-hoc* tests were not significant for mean turning speed, peak turning speed was significantly faster in SBCR at RET (p_−FDR_ = 0.019). Importantly, no within-group increases in either mean or peak turning speed were found in TBT.

#### Cognitive Tasks

No significant interactions were found for Stroop response time while walking. However, secondary outcomes of Stroop performance while walking did show significant interactions between SBT and TBT, particularly Stroop response time variability (*p* < 0.001), accuracy (*p* < 0.001) and performance index (*p* < 0.001). SB75 (p_−FDR_ = 0.016) and SB50 (p_−FDR_ < 0.001) showed greater reductions in response time variability at RET compared to TBT. Although TBT showed higher accuracy at POST compared to SB75, SB50, and SBCR (all p_−FDR_ < 0.01), differences were lost at RET. A similar pattern was seen for performance index with significant higher values in TBT at POST compared to SB50 (p_−FDR_ = 0.004), while at RET, SB75 showed higher performance index compared to TBT (p_−FDR_ = 0.031).

**Figure 2:**
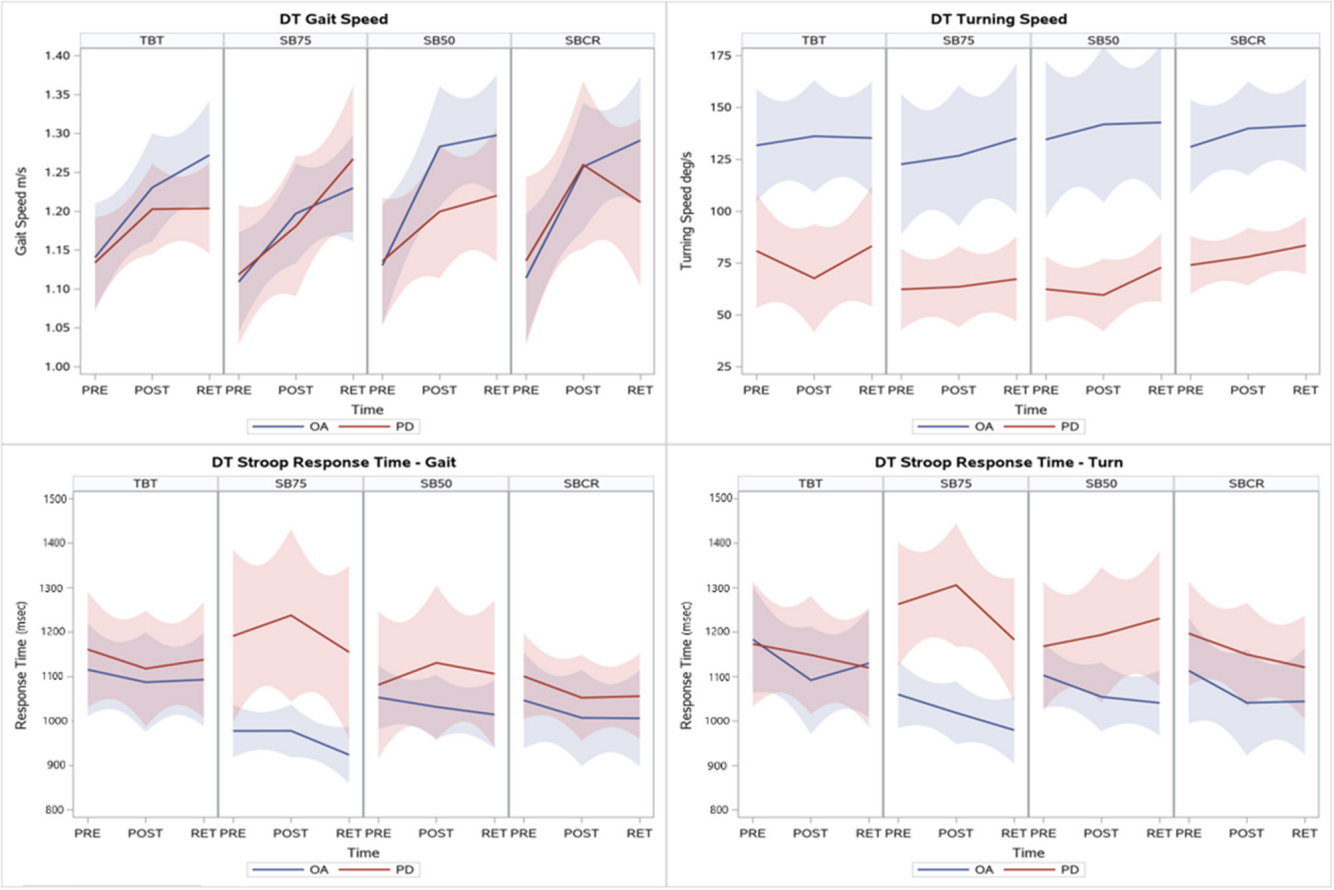
Locally estimated scatterplot smoothing (LOESS) regression lines and 95% confidence intervals from PRE to POST and RET for the primary outcomes fitted separately for OA and PD+FOG within each training group. In the full sample analysis combining data from OA and PD+FOG, increase in dual-task gait speed and decrease in stroop response time while turning were seen in all groups, while only SBT groups increased their dual-task turning speed and decreased stroop response time while walking. Similar results were seen in the PD+FOG sub-group, although with smaller effect sizes.

No significant interactions between TBT and SBT were found for cognitive task performance while turning, as all groups showed similar reductions on Stroop response time and increase in performance index at RET. Progression within OA and PD+FOG on primary outcomes is shown in Figure 2.

### Sensitivity Analysis in PD+FOG

#### Motor Tasks

As with the full sample analyses, significant Group^*^Time interactions were found between SBT and TBT on DT stride length (*p* = 0.006), with *post-hoc* tests revealing that SBCR had larger increase in stride length at POST (p-_FDR_ = 0.011). Differences between SBT and TBT were not significant at RET as all groups showed similar within-group increments.

Similarly, significant Group^*^Time interactions were found for DT mean (*p* = 0.039) and peak turning speed (*p* = 0.046). *Post-hoc* tests at RET showed a trend toward significantly faster turning in all SBT groups compared to TBT (all p-_FDR_ ≤ 0.1). Additionally a significant interaction effect was found for mean turning speed variability (*p* = 0.037), with TBT showing a larger decrease in mean turning speed variability compared to SB50 at POST (p-_FDR_ = 0.046). These differences were not maintained at RET.

#### Cognitive Tasks

Stroop accuracy (*p* = 0.016) and performance index (*p* = 0.054) showed significant interactions between TBT and SBT, however *post-hoc* tests were not significant.

### Comparison of SBT Within-Group Effect Sizes at RET

**Figure 3:**
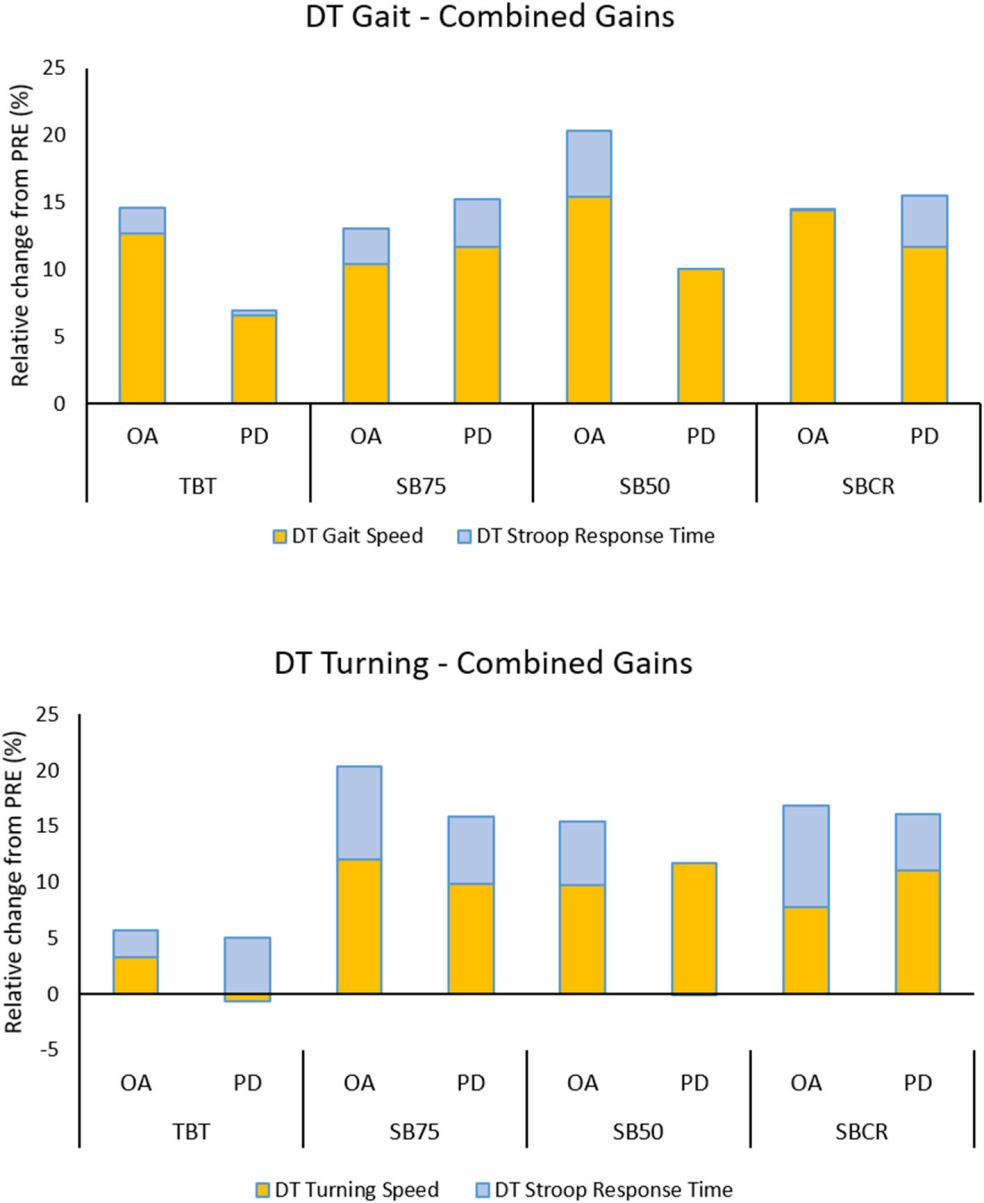
Investigation of trade-off between motor and cognitive gains. Relative change from PRE for motor and cognitive performance while walking (upper panel) and turning (lower panel), shown separately for training and OA and PD+FOG sub-groups. Raw change scores from PRE to RET were calculated and normalized to % PRE value. Median values for each training group and sub-group is shown on a stacked bar chart to show the additive or competitive effects of gains on the motor vs. the cognitive task. Stroop change scores were inverted, so that positive scores are better. Combined effects were consistently larger for SBT groups for DT turning, in OA as well as PD+FOG. Some tradeoff between motor and cognitive gains is seen while turning in persons with PD in TBT and SB50 groups.

In the full sample analysis, effect sizes at RET were largest for SB50 (d_w_ = 2.84) for DT gait speed, while SBCR had largest effect size on mean DT turning speed (d_w_ = 0.9), and SB75 showed largest effect sizes on Stroop response time while walking (d_w_ = −1) and turning (d_w_ = −1.19). When looking only at the sub-group of PD+FOG, effect sizes at RET were largest for SB75 for DT gait speed (d_w_ = 1.37) and Stroop response time while walking (d_w_ = −1), while SBCR showed the largest effect size for mean DT turning speed (d_w_ = 0.77) and Stroop response time while turning (d_w_ = −0.97). Exploration of the relative improvement (median group % change) on primary outcomes from PRE to RET showed that trade-off between improvements on motor and cognitive performance were only seen for DT turning in PD+FOG in TBT and SB50 (Figure 3).

## Discussion

In this single-session training study, we investigated the effects of SBT and TBT on DT walking and DT turning in PD+FOG and healthy OA. In line with our hypotheses, both SBT and TBT led to improvements on DT gait speed, while only SBT improved DT turning speed. Improvements on the Stroop task while walking and turning were seen in all groups, in contrast to our expectations. However, in PD+FOG, improvements in stoop response time while turning may have come at the expense of motor improvements in the TBT group. Further, improvements in DT gait speed and Stroop performance while walking were larger in SBT compared to TBT. This transfer of SBT effects to DT walking and turning, with moderate to large effect sizes, highlights the promising role of SBT to improve DT performance in PD+FOG as well as in OA.

In their review of the effect of treadmill training in PD, Mehrholz and colleagues found it to be a robust tool to improve single-task gait, particularly gait speed and stride length (51). We were able to replicate these results under dual-task conditions (improvement in DT gait speed from PRE to RET in TBT: 0.09 m/s, 95% CI: 0.06–0.12, Mehrholz: 0.09 m/s, 95% CI: 0.03–0.14), and additionally showed that SBT led to even larger effects on these outcomes (improvement from PRE to RET in SB50: 0.16 m/s, 95% CI: 0.12–0.19), in the range of clinically meaningful improvements (52). The fact that these effects were clearly visible when attention was diverted, suggests that gait automaticity was improved by the training. The mechanism for SBT to induce stronger automatization of gait compared to TBT is unknown. Treadmill walking is externally guided as movement is driven by the belts. Externally guided movement is thought to rely strongly on cerebellar input within cerebellar-thalamo-ventral premotor networks (53). Indeed, after a 6-month treadmill training program, stroke patients showed increased activation in the cerebellum and midbrain during movement of their paretic leg, which was associated with gains in walking velocity (54). During our training, both TBT and SBT would benefit from recruitment of this network, freeing up frontal executive resources (55) for learning related processes (56). In this boosted learning environment, split-belt demands may lead to additional gains, through other mechanisms that were at play. One of these mechanisms may be imposing sensory prediction error repeatedly through these abnormal split-belt gait patterns that required motor adaptation, in which the cerebellum also plays an important role (57). Evidence supporting this mechanism comes from two studies that showed that cerebellar damage impairs SBT adaptation (58), and that cerebellar modulation with non-invasive stimulation influences spatial adaptation during SBT (59). However, the exact neurophysiological mechanism on how the relationship between varying sensory manipulations and motor responses strengthens the learning circuits within the cerebellum are still largely illusive (57). Taken together, we surmise that adaptations driven by sensory prediction error based training with SBT recruits the cerebellum and cerebellar locomotor region additionally compared to TBT, leading to larger spatial gait effects seen here.

In this study, these adaptations led to a greater increase in gait speed, stride length, and turning speed, possibly by increasing step length on the side that was slowed down. These measures are highly related, recently being shown to load on the same movement domain and to be strongly associated with attention and executive function (60). Based on the attentional capacity theory of dual-task interference, we expected that improved automaticity on the motor task would be accompanied by improved cognitive task performance, as cognitive resources would be freed from gait control. In this cohort, all groups apart from SB75 had average MoCA scores below the cutoff for mild cognitive impairment. This may suggest a diminished cognitive reserve and limited capacity for improvement. Despite this, besides response time itself, improvements on cognitive measures such as the performance index and response time variability were found. Response time variability measures the construct of fluctuating attention, which deteriorates early in PD and may be a marker of PD dementia (61). These results indicate that SBT would be beneficial even for persons with cognitive impairment, and may slow down cognitive decline. Importantly, these improvements on the cognitive task did not come at the expense of, but rather in addition to the improvements on the motor tasks (recall Figure 3). Overlap in the neural resources responsible for cognitive set-shifting and motor adaptation, may have resulted in cognitive facilitation (62), particularly while turning. One contradictory cognitive result was that TBT showed larger improvements compared to SBT on accuracy on the Stroop while walking post-training. The lack of improvement in Stroop accuracy for SBT may be explained by a difference in intensity of the training, as deterioration in accuracy of cognitive tasks has been reported after acute intermediate intensity exercise (63). Although we did not find differences on self-report measures of perceived intensity, the limping gait during SBT likely had higher energy costs than regular gait on the tied-belts. Further, these seemingly negative effects were transient as they were not detectable at retention. Moreover, performance index which takes into account the speed-accuracy tradeoff, was significantly more improved in SBT at retention. Overall, the SBT effects seen were very positive, both for the motor as well as the cognitive effects.

The effects seen in the PD sub-group alone were promising, although they seemed to suffer from the motor learning deficits known to affect PD+FOG in particular (64). In spite of this, differences between SBT and TBT were found on DT stride length and peak DT turning speed. A key difference noticed in the sensitivity analyses, was that SB50 was less effective in PD than in OA (Supplementary Table 1 and Figures 2, 3). Analysis of adaptations during the training session may provide insights into whether PD+FOG were able to adapt to the large belt-speed difference, and the large asymmetry induced. Further, interpreting the large error without any explicit feedback about the direction in which, or degree to which to adapt, can be particularly challenging for PD+FOG (65). These failings of SB50 were contrary to our expectations on the secondary aim. Effects of SB75 and SBCR seemed consistent, in both the full sample and PD+FOG sub-group. Of note, gains from the training were often larger at retention than at post-training (Table 2 and Figure 2), indicating that the training triggered offline consolidation on these tasks. Aerobic exercise has been shown to improve offline consolidation of a novel task in PD (66), and we expect that similar mechanisms were at work here for relearning previously trained tasks. These effects were particularly evident for SB75, which in contrast to the more explicit SB50 and SBCR conditions, may have relied on implicit learning processes due to the almost imperceptible belt speed differences (67). On the other hand, the effects of fatigue on post-training measurements cannot completely be ruled out, after the training and the extensive testing pre-training.

### Limitations and Recommendations

This study, to our knowledge, is the first clinical study to investigate the effectiveness of treadmill training on dual-task outcomes specifically, besides presenting proof of the potential of SBT for improving dual-tasking in PD and OA. The study was slightly underpowered to detect training differences within the sub-group of PD+FOG alone. Therefore, we pooled OA and PD for the main analysis, and performed a sensitivity analysis in the PD+FOG. Apart from SB50, results for the remaining groups were very similar in both analyses. These results allayed concerns of a differential response in OA and PD, in line with previous work (21). Future work could utilize a repeated training design to improve effect sizes and power to detect differences in the PD+FOG. Another limitation of the study is that it did not include a broader sample of people with PD. While this limits the generalizability of these results, given the motor learning difficulties in PD+FOG (64), effects would likely extend to people without FOG. While interpreting the effects of SBT on turning outcomes, it is of some import to note that test-retest reliability of these turning metrics with or without dual-tasking, and their clinical relevance have not yet been established. Longer term follow-up in free-living conditions would be required to investigate transfer to daily life, as laboratory based turning may not reflect turning at home (68). Further, this study used preferred dual-task gait speed as a primary outcome. Inter-individual interpretation of the “as usual” instruction may have led to some participants measuring closer to capacity than others. On the other hand, “as fast as possible” was the instruction given for the turning in place task, which led to a similar pattern of improvements from PRE, downplaying concerns over the influence of this instruction. Future studies using gait speed as an outcome should ensure that they standardize instructions across tasks to capture true increases in dual-task capacity rather than just performance.

Although this study presents some aspects of the effects of SBT on dual-tasking with moderate to large effect sizes, these are only the immediate and short-term effects. How these would translate to dual-tasking in the daily life environment and the impact on falls and FOG should be the subject of future longer term training studies. Based on the effect sizes, SBCR would be preferred to improve dual-task turning, while SB75 might be preferred for its low challenge and stable effects, particularly in participants with cognitive decline such as PD+FOG. Alternatively, providing relevant feedback while training may assist persons with PD to adapt to SB50 similarly to OA, which may also improve outcomes. These questions are yet to be answered before personalized prescription of SBT for dual-tasking will be made possible.

## Conclusion

This study investigated the effects of single-session split-belt treadmill training, compared to regular treadmill training, on dual-task walking and turning in people with PD with FOG and healthy OA. We found that split-belt training led to significantly larger effects on dual-task gait compared to tied-belt training and its effects also transferred to a dual-task turning task. Further, most of these training effects were stronger when tested the following day, suggesting offline consolidation. These results provide initial evidence of the potential for SBT to improve dual tasking during complex gait in PD as well as OA. Longer training and follow-up studies are required to further explore the impact of this training on daily function and disease burden.

## Data Availability Statement

The datasets presented in this article are not readily available because of ethical and privacy reasons. Requests to access the datasets should be directed to Nicholas D'Cruz, nicholas.dcruz@kuleuven.be.

## Ethics Statement

The studies involving human participants were reviewed and approved by Ethics Committee for Research UZ/KU Leuven and Ethics Committee of the University Hospital Schleswig-Holstein Kiel. The patients/participants provided their written informed consent to participate in this study.

## Author Contributions

ND'C: research project execution, data acquisition, processing, statistical analysis design, execution, and manuscript writing of the first draft. JS: research project execution, data acquisition, processing, and manuscript review and critique. PG and FH: research project execution, data acquisition, and manuscript review and critique. CS and AN: research project conception, organization, research project execution, statistical analysis review and critique, and manuscript review and critique. All authors: contributed to the article and approved the submitted version.

## Conflict of Interest

The authors declare that the research was conducted in the absence of any commercial or financial relationships that could be construed as a potential conflict of interest.
