# Supplementary material for: Short-Term Effects of Single-Session Split-Belt Treadmill Training on Dual-Task Performance in Parkinson's Disease and Healthy Elderly
Source: Front Neurol. 2020 Sep 30;11:560084. doi: 10.3389/fneur.2020.560084 (PMC7554617; doi:10.3389/fneur.2020.560084)
Supplement: Supplementary file 1 [file Table_1.docx]

|  | **Outcome** | **Time** | **TBT** | | **SB75** | | **SB50** | | **SBCR** | | **Within** | **Group*Time** | **Group*Time**  *Post-hoc* |
| --- | --- | --- | --- | --- | --- | --- | --- | --- | --- | --- | --- | --- | --- |
|  |  |  | *Estimate (95% CI)* | *d_w_* | *Estimate (95% CI)* | *d_w_* | *Estimate (95% CI)* | *d_w_* | *Estimate (95% CI)* | *d_w_* | *p* | *p* |  |
|  | DT Gait Speed (m/s) | PRE - POST | 0.07 (0.00 - 0.13) | 0.70^†^ | 0.07 (0.00 - 0.13) | 0.71^‡^ | 0.06 (-0.0 - 0.16) | 0.54^†^ | 0.11 (0.06 - 0.17) | 1.35^‡^ | **<.001** | 0.097 |  |
| GAIT |  | PRE - RET | 0.07 (0.03 - 0.11) | 1.20^‡^ | 0.13 (0.06 - 0.20) | 1.37^‡^ | 0.09 (0.00 - 0.17) | 0.80^‡^ | 0.07 (0.00 - 0.14) | 0.70^‡^ |  |  |  |
|  | DT Gait Speed CV (%) | PRE - POST | -1.6 (-7.4 - 4.10) | -0.21 | 1.89 (-0.3 - 4.12) | 0.38 | -2.6 (-8.2 - 2.90) | -0.32 | -1.6 (-5.8 - 2.58) | -0.25 | **<.001** | 0.186 |  |
|  |  | PRE - RET | -0.7 (-3.5 - 2.12) | -0.19^†^ | -4.3 (-7.9 - -0.8) | -0.86^†^ | -5.4 (-8.8 - -2.1) | -0.77^†^ | -3.3 (-7.5 - 0.79) | -0.49^†^ |  |  |  |
|  | DT Gait Stride Length (mm) | PRE - POST | 12.1 (-38 - 62.7) | 0.13 | 10.1 (-46 - 66.6) | 0.12^†^ | 33.5 (-56 - 123) | 0.28^†^ | 93.5 (51.8 - 135) | 1.12^‡^ | **<.001** | **0.006** | SBCR > TBT |
|  |  | PRE - RET | 37.3 (3.79 - 70.8) | 0.79^‡^ | 107 (58.4 - 155) | 1.52^‡^ | 63.0 (-6.4 - 132) | 0.68^‡^ | 45.2 (-25 - 116) | 0.42^†^ |  |  | NS |
|  | DT Gait Stride Length CV (%) | PRE - POST | -0.4 (-6.4 - 5.48) | -0.06 | 2.04 (-0.2 - 4.33) | 0.54 | -2.7 (-7.4 - 1.97) | -0.38 | -2.7 (-7.0 - 1.45) | -0.42 | **0.001** | 0.068 |  |
|  |  | PRE - RET | 0.12 (-2.3 - 2.55) | 0.03 | -3.8 (-6.7 - -0.9) | -0.93^†^ | -3.9 (-8.4 - 0.62) | -0.62^†^ | -1.8 (-7.0 - 3.26) | -0.24* |  |  |  |
|  | DT Gait Step Width (mm) | PRE - POST | -4.0 (-17 - 9.31) | -0.22 | -2.3 (-4.8 - 0.11) | -0.60 | -2.5 (-13 - 8.17) | -0.18 | -3.2 (-7.7 - 1.22) | -0.47 | 0.273 | 0.778 |  |
|  |  | PRE - RET | 2.75 (-8.1 - 13.6) | 0.19 | -4.4 (-12 - 3.43) | -0.39 | -11 (-22 - -1.5) | -0.81 | 2.47 (-10 - 15.1) | 0.12 |  |  |  |
|  | DT Gait Step Width CV (%) | PRE - POST | 1.38 (-2.8 - 5.62) | 0.23 | 2.75 (-3.6 - 9.12) | 0.30 | -4.9 (-13 - 4.07) | -0.41 | -3.0 (-8.8 - 2.82) | -0.29 | **0.027** | 0.120 | NS |
|  |  | PRE - RET | -3.0 (-9.9 - 3.89) | -0.33 | -1.2 (-8.6 - 6.14) | -0.11 | 1.92 (-2.2 - 6.10) | 0.34 | -3.4 (-9.7 - 2.90) | -0.31 |  |  | NS |
| TURNING | DT Turning Speed (°/s) | PRE - POST | -10 (-33 - 12.8) | -0.34 | 5.58 (-8.2 - 19.4) | 0.28 | -3.9 (-18 - 10.8) | -0.20 | 5.69 (-5.6 - 17.0) | 0.32 | **0.002** | **0.039** | NS |
|  |  | PRE - RET | -6.4 (-22 - 9.18) | -0.30 | 7.37 (-1.7 - 16.5) | 0.55^†^ | 7.81 (-3.6 - 19.2) | 0.50* | 10.3 (1.59 - 19.1) | 0.77^†^ |  |  | NS |
|  | DT Turning Speed CV (%) | PRE - POST | -4.1 (-6.3 - -1.9) | -1.23^†^ | 0.20 (-4.3 - 4.81) | 0.03 | 6.43 (-3.1 - 15.9) | 0.54 | 0.37 (-6.5 - 7.25) | 0.03 | **0.014** | **0.037** | TBT < SB50 |
|  |  | PRE - RET | -0.2 (-5.0 - 4.53) | -0.04 | 0.81 (-8.3 - 9.95) | 0.06 | -0.3 (-4.0 - 3.38) | -0.06 | -1.5 (-5.0 - 1.97) | -0.26 |  |  |  |
|  | DT Peak Turning Speed (°/s) | PRE - POST | -9.4 (-48 - 29.3) | -0.18 | 16.8 (-1.8 - 35.6) | 0.63^†^ | -4.1 (-23 - 15.2) | -0.16 | 3.76 (-9.4 - 16.9) | 0.18 | **0.006** | **0.046** | NS |
|  |  | PRE - RET | -2.4 (-12 - 7.56) | -0.23 | 10.0 (-4.5 - 24.5) | 0.48^†^ | 11.2 (-5.0 - 27.5) | 0.51^†^ | 14.9 (3.78 - 26.0) | 0.87^†^ |  |  | NS |
|  | DT Peak Turning Speed CV (%) | PRE - POST | -0.0 (-0.0 - 0.02) | -0.19 | 0.01 (-0.0 - 0.03) | 0.24 | 0.02 (-0.0 - 0.06) | 0.27 | 0.00 (-0.0 - 0.03) | 0.14 | 0.812 | 0.653 |  |
|  |  | PRE - RET | -0.0 (-0.0 - 0.05) | -0.15 | 0.02 (-0.0 - 0.09) | 0.29 | 0.00 (-0.0 - 0.02) | 0.10 | 0.00 (-0.0 - 0.03) | 0.03 |  |  |  |
|  | DT Turning Jerkiness (m2/s5 * 100) | PRE - POST | -2.3 (-8.9 - 4.16) | -0.26 | -1.7 (-5.6 - 2.07) | -0.31 | 4.47 (-7.3 - 16.2) | 0.27 | 2.06 (-2.0 - 6.21) | 0.32 | 0.333 | 0.322 |  |
|  |  | PRE - RET | 5.98 (-4.1 - 16.1) | 0.50 | -0.9 (-3.9 - 2.07) | -0.21 | 0.28 (-6.0 - 6.67) | 0.03 | 1.43 (-2.0 - 4.95) | 0.25 |  |  |  |
| GAIT STROOP | Gait Stroop Response Time (ms) | PRE - POST | -4.1 (-109 - 26.3) | -0.46 | 0.00 (-77 - 77.2) | 0.00 | 2.58 (-27 - 79.5) | 0.37 | -3.3 (-90 - 24.4) | -0.38 | **0.006** | 0.268 |  |
|  |  | PRE - RET | -2.1 (-75 - 32.8) | -0.29 | -3.6 (-61 - -10) | -1.00^†^ | 1.19 (-75 - 99.9) | 0.10 | -3.5 (-79 - 8.84) | -0.51 |  |  |  |
|  | Gait Stroop Response Time CV (%) | PRE - POST | -2.0 (-4.2 - 0.16) | -0.64 | -2.2 (-4.9 - 0.39) | -0.48* | -1.7 (-5.5 - 2.00) | -0.42 | -0.7 (-2.7 - 1.35) | -0.17 | **0.043** | 0.239 |  |
|  |  | PRE - RET | 0.64 (-3.2 - 4.54) | 0.11 | -2.2 (-4.5 - 0.05) | -0.56* | 1.33 (-2.3 - 4.98) | 0.41 | -0.5 (-2.4 - 1.43) | -0.17 |  |  |  |
|  | Gait Stroop Accuracy (%correct) | PRE - POST | 5.83 (0.48 - 11.1) | 0.76 | 4.69 (0.95 - 8.43) | 0.88 | -4.9 (-13 - 3.62) | -0.59 | 1.32 (-6.0 - 8.65) | 0.09 | **0.004** | **0.016** | NS |
|  |  | PRE - RET | -0.8 (-5.0 - 3.26) | -0.15 | 4.78 (0.41 - 9.15) | 0.74 | 1.46 (-15 - 18.1) | 0.09 | 2.24 (-5.3 - 9.84) | 0.14 |  |  | NS |
|  | Gait Stroop Performance Index (%/s) | PRE - POST | 7.43 (-0.2 - 15.1) | 0.72 | 5.91 (0.00 - 11.8) | 0.70 | -6.7 (-14 - 1.33) | -0.68 | 6.47 (-4.6 - 17.5) | 0.38 | **0.003** | 0.054 |  |
|  |  | PRE - RET | 1.25 (-5.7 - 8.22) | 0.13 | 6.64 (2.35 - 10.9) | 1.07^†^ | -0.0 (-17 - 17.3) | -0.00 | 6.43 (-3.4 - 16.3) | 0.40 |  |  |  |
| TURNING STROOP | Turning Stroop Response Time (ms) | PRE - POST | -14 (-43 - 14.7) | -0.38^†^ | 4.62 (-75 - 84.8) | 0.03 | -2.1 (-98 - 94.4) | -0.01 | -59 (-112 - -5.3) | -0.57^†^ | **<.001** | 0.260 |  |
|  |  | PRE - RET | -4.9 (-110 - 12.7) | -0.58^†^ | -53 (-92 - -15) | -0.86^†^ | 26.9 (-73 - 127) | 0.20 | -62 (-104 - -20) | -0.97^†^ |  |  |  |
|  | Turning Stroop Response Time CV (%) | PRE - POST | 1.29 (-2.5 - 5.17) | 0.26 | 0.17 (-4.7 - 5.10) | 0.02 | -0.5 (-3.3 - 2.20) | -0.16 | -1.6 (-5.2 - 1.92) | -0.29 | 0.530 | 0.926 |  |
|  |  | PRE - RET | 0.54 (-3.9 - 5.01) | 0.10 | -0.6 (-3.5 - 2.13) | -0.16 | 0.15 (-5.7 - 6.03) | 0.02 | -2.0 (-5.4 - 1.45) | -0.38 |  |  |  |
|  | Turning Stroop Accuracy (%correct) | PRE - POST | -3.6 (-11 - 4.19) | -0.33 | 3.55 (-2.9 - 10.0) | 0.38 | -0.6 (-8.1 - 6.98) | -0.06 | -3.3 (-11 - 4.86) | -0.26 | 0.114 | 0.782 |  |
|  |  | PRE - RET | 1.63 (-1.7 - 5.03) | 0.38 | 4.68 (-3.4 - 12.7) | 0.40 | 4.46 (-7.1 - 16.1) | 0.28 | 4.11 (-3.0 - 11.3) | 0.28 |  |  |  |
|  | Turning Stroop Performance Index (%correct/s) | PRE - POST | -1.5 (-9.7 - 6.70) | -0.13 | 5.08 (-0.9 - 11.1) | 0.58 | -1.0 (-10 - 8.43) | -0.08 | -0.4 (-9.3 - 8.52) | -0.03 | **0.014** | 0.952 |  |
|  |  | PRE - RET | 5.05 (-2.4 - 12.6) | 0.50^†^ | 6.89 (-1.4 - 15.2) | 0.57^†^ | 1.09 (-12 - 14.8) | 0.05 | 7.83 (0.67 - 14.9) | 0.66^†^ |  |  |  |

**Supplementary Table 1.** Estimated change for each training group (PD+FOG only) from PRE to POST and PRE to RET, along with 95% Confidence Intervals (CI) and within-group effect sizes (d_w_ from t statistic). Effect size values are reported in the direction of the raw data, not in the direction of expected improvement. F-test probability values for within and between group effects are also reported, along with post-hoc tests for which multiple comparison correction of within group effects was performed with the False Discovery Rate method (FDR). Between group post-hoc tests did not survive multiple comparison correction, so uncorrected comparisons are reported (p < 0.05). Statistical significance indicated by * p_FDR_ < 0.1, ^†^ p_FDR_ < 0.05, ^‡^ p_FDR_ < 0.001 or **Bold** probability values.
